# Single-Stage Posterior Approach for a Neglected Severe Kyphotic Spine With a Three-Year Follow-Up

**DOI:** 10.7759/cureus.32459

**Published:** 2022-12-12

**Authors:** Mohamed Razzan Rameez, Mohd Hisam Muhamad Ariffin

**Affiliations:** 1 Orthopedics and Traumatology, Hospital Universiti Kebangsaan Malaysia, Kuala Lumpur, MYS; 2 Spine Surgery, Universiti Kebangsaan Malaysia Medical Centre, Kuala Lumpur, MYS

**Keywords:** single staged surgery, kyphoscoliosis, posterior approach, deformity correction, neglected kyphosis

## Abstract

Neglected spinal tuberculosis with severe kyphosis is uncommon. Spinal tuberculosis can present with back pain, neurological deficits, cold abscesses, or severe deformities. Diagnosis is made using laboratory, imaging, and tissue studies. Management can be done medically or surgically. As neurological deficits or severe deformities worsen, most surgeons prefer a surgical option. There have been different opinions regarding the surgical approach for spinal tuberculosis. This is a case of neglected spinal tuberculosis with severe kyphotic deformity treated with single-stage posterior instrumentation and fusion without any complications within in a three-year follow-up period.

## Introduction

Severe kyphosis can be potentially life-threatening, causing cardiopulmonary and digestive complications, spinal imbalance, neurological deficits, and decreased quality of life. One common cause of severe gibbus deformity is untreated spinal tuberculosis which infects more than 25% of the world’s population, of which 85% to 95% experience latent infections. The spine is the most common site of skeletal tuberculosis [1,2].

Spinal kyphosis is commonly due to the collapse of the anterior column of the spinal vertebral column involving the adjacent vertebral body. Spinal tuberculosis has been easily neglected due to its insidious presentation with cold abscesses, neurological deficits, and/or severe kyphosis. We report a case of neglected spinal tuberculosis with severe kyphosis, managed surgically with a single-stage posterior approach.

## Case presentation

A 33-year-old female presented with bilateral lower limb numbness with difficulty ambulating associated with occasional back pain with a progressive deformity for the past year. However, due to her socio-economic status, she presented late. She did not have any constitutional symptoms. On examination, the Medical Research Council grading of her upper and lower limb muscle power was normal with exaggerated reflexes and intact sensation.

**Figure 1:**
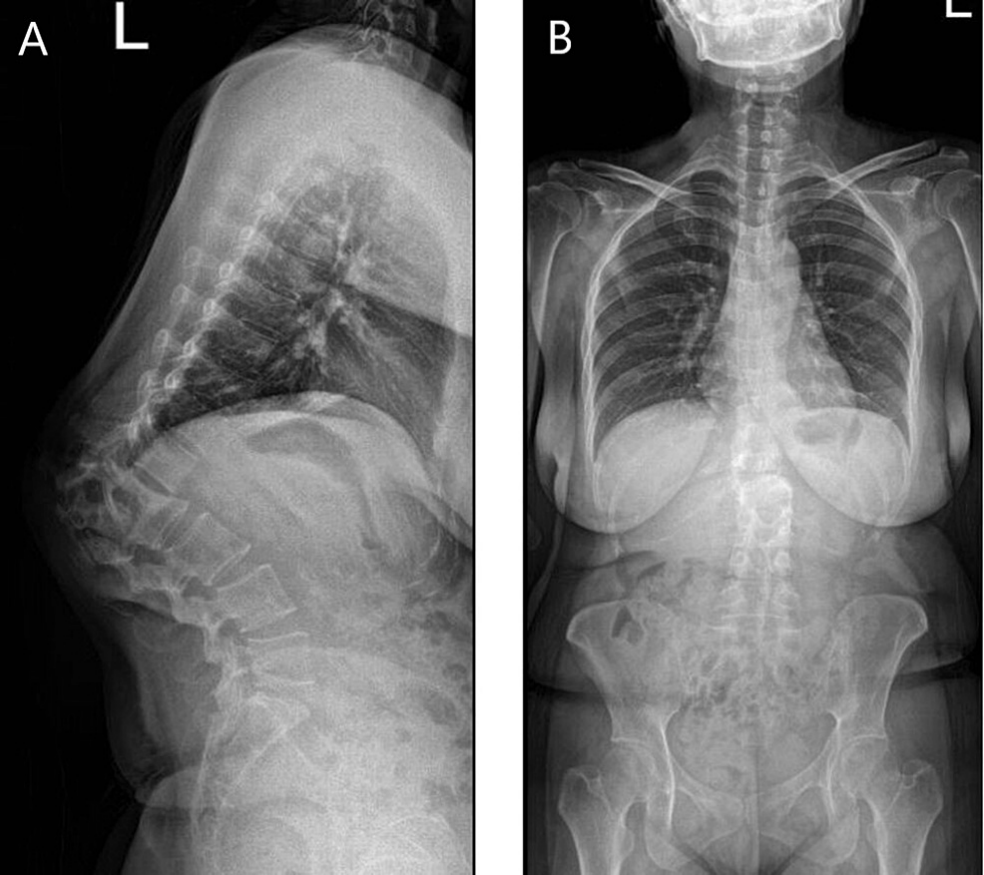
Preoperative lateral (A) and anteroposterior (B) whole-spine radiographs of the patient's severe kyphosis.

**Figure 2:**
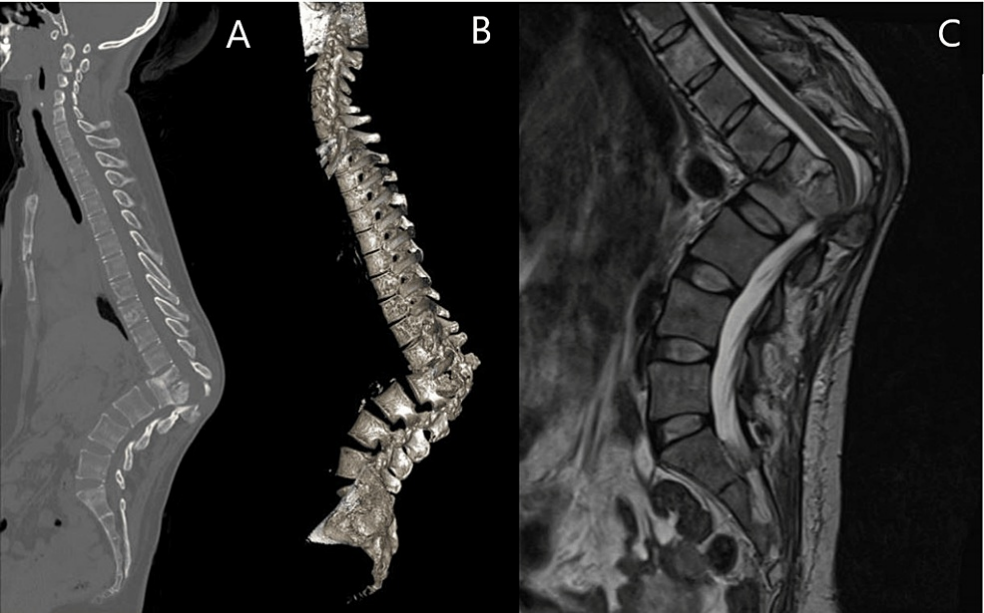
Preoperative CT (A) and 3D CT reconstruction (B) showing T12–L2 vertebrae with acute-angle kyphosis, and T2-weighted MRI (C) showing spondylodiscitis of the T12–L2 vertebrae with paraspinal collection.

Laboratory investigations revealed an increased erythrocyte sedimentation rate and a positive Mantoux test. Spinal radiography and computed tomography showed severe kyphosis at 87.9° from T12 to L2 due to the collapse of the anterior vertebral column (Figures 1-2). The patient had a sagittal imbalance with a sagittal vertical axis deviation of 6.8 cm. Whole-spine magnetic resonance imaging revealed spondylodiscitis at the T12-L2 level with an anterior paraspinal collection.

The patient was started on anti-tuberculosis medications and was counseled for surgical intervention in view of her severe kyphosis affecting her daily activities and worsening further.

She underwent single-stage posterior instrumentation and fusion of the T8-L5 vertebra with vertebrectomy. Intraoperatively, her left T12 nerve root was sacrificed to access and debride the caseous tissue (Video 1). Vertebrectomy was done at the apex of the deformity and a hydrolift cage was inserted. A cantilever maneuver was performed on prebent rods, and the spinal deformity was corrected based on post-deformity intraoperative neuromonitoring.

**Video 1:** Intraoperative video of severe kyphosis deformity.

**Figure 3:**
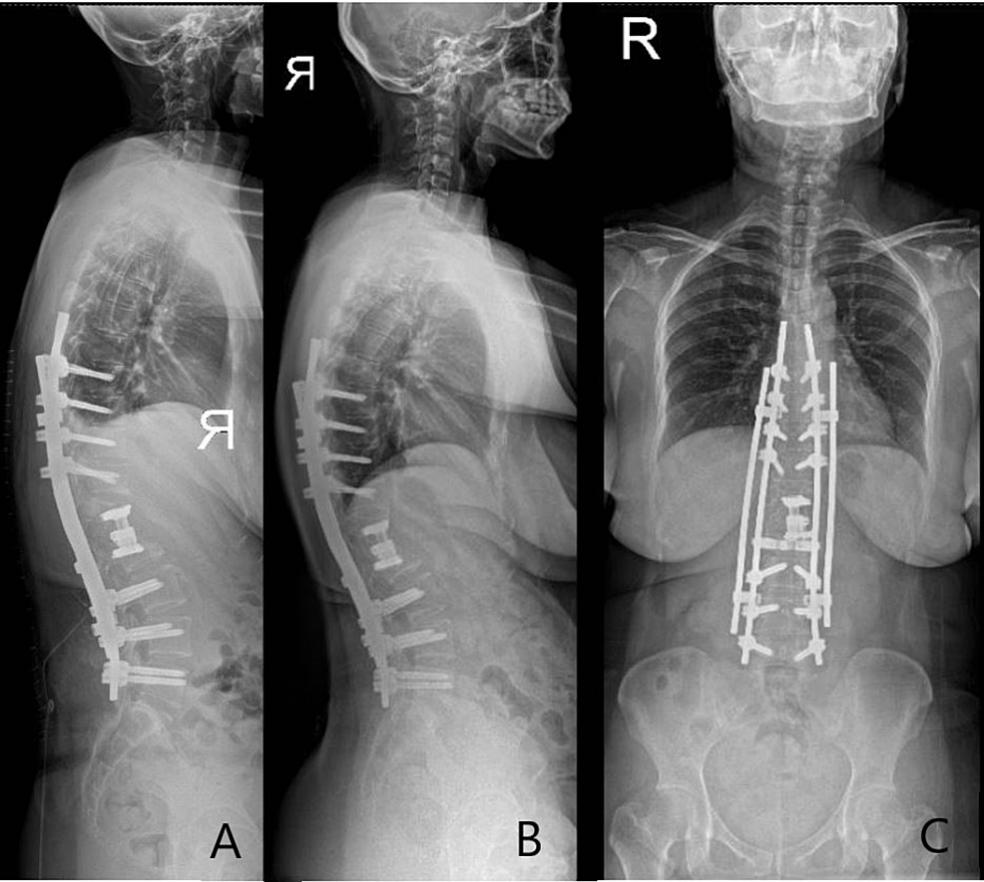
Immediate postoperative standing lateral whole-spine radiograph with posterior instrumentation and cage insertion (A). Three-year postoperative lateral (B), and anteroposterior (C) whole-spine radiographs.

**Figure 4:**
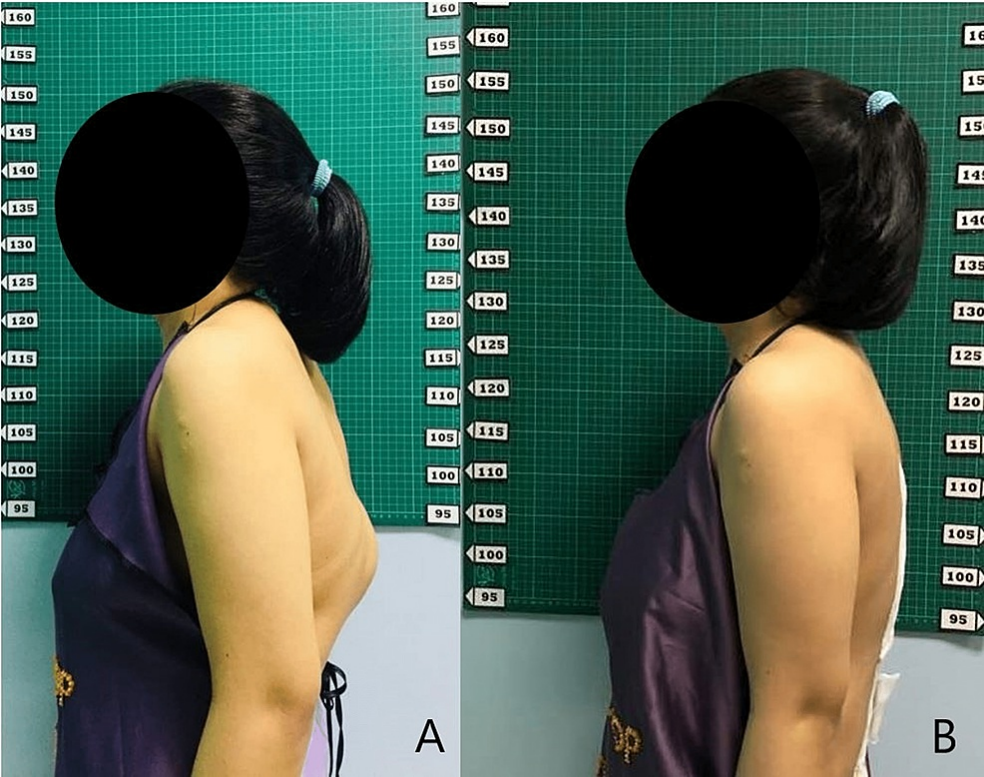
Preoperative (A) and postoperative (B) standing lateral views of the patient.

Postoperatively, the numbness improved with no neurological deficits. Within three years the patient was able to do normal chores at home independently with no active issues (Figures 3-4)

## Discussion

Severe adulthood kyphosis is mostly due to neglected spinal tuberculosis which presents with secondary symptoms such as weakness, numbness, breathing difficulties, and paraplegia [3]. Neurological complications of spinal tuberculosis are around 10% and can go up to 43% if left untreated [4].

Standard protocols for managing spinal tuberculosis have been changing with new technological improvements and research. Anti-tuberculosis medications are still the gold standard in all tuberculosis types and prevent drug resistance and disease relapse. Hence, we started the patient on anti-tuberculosis medication before any surgical intervention [1].

Our patient presented with late severe kyphosis with bilateral lower limb numbness thereby warranting surgical intervention. Multiple surgical approaches have been practiced, such as anterior, posterior, and combined anterior-posterior approaches, with continuing debates on which approach is superior.

The anterior approach gives more anterior exposure, allowing the easy release of the anterior spinal column due to the collapse. However, the visceral and large vessels surrounding the surgical field may be injured. The approach can also lead to kyphosis as good stability anteriorly might not be achieved [2]. The traditional combined approach provides better fixation; however, it has high complication rates in terms of blood loss, longer operative time, and large surgical soft tissue trauma [1].

Posterior instrumentation and fusion are commonly used in treating severe kyphosis [2]. It shortens the surgical duration which decreases the postoperative complications and trauma and provides good posterior and anterior spinal stability. However, this procedure comes with some postoperative complications such as spinal cord injury, nerve root damage during manipulation, and muscle weakness. We proceeded with a single-stage posterior approach for the patient and achieved anterior release via this approach.

The surgery allowed for circumferential decompression and release. An expandable cage (Hydrolift Aesculap AG, B Braun, Melsungen, Germany) was inserted posteriorly to prevent shortening and buckling of the cord at the conus, which may cause neurological deficits. Expanding the cage lengthens the anterior column, restores the normal length of the cord, and serves as a fulcrum for the subsequent cantilever maneuver for complete anatomical correction of the sagittal balance. Combining the posterior instrumentation and the cantilever maneuver gives a strong corrective force, in which anterior resection is not needed. [5]

**Figure 5:**
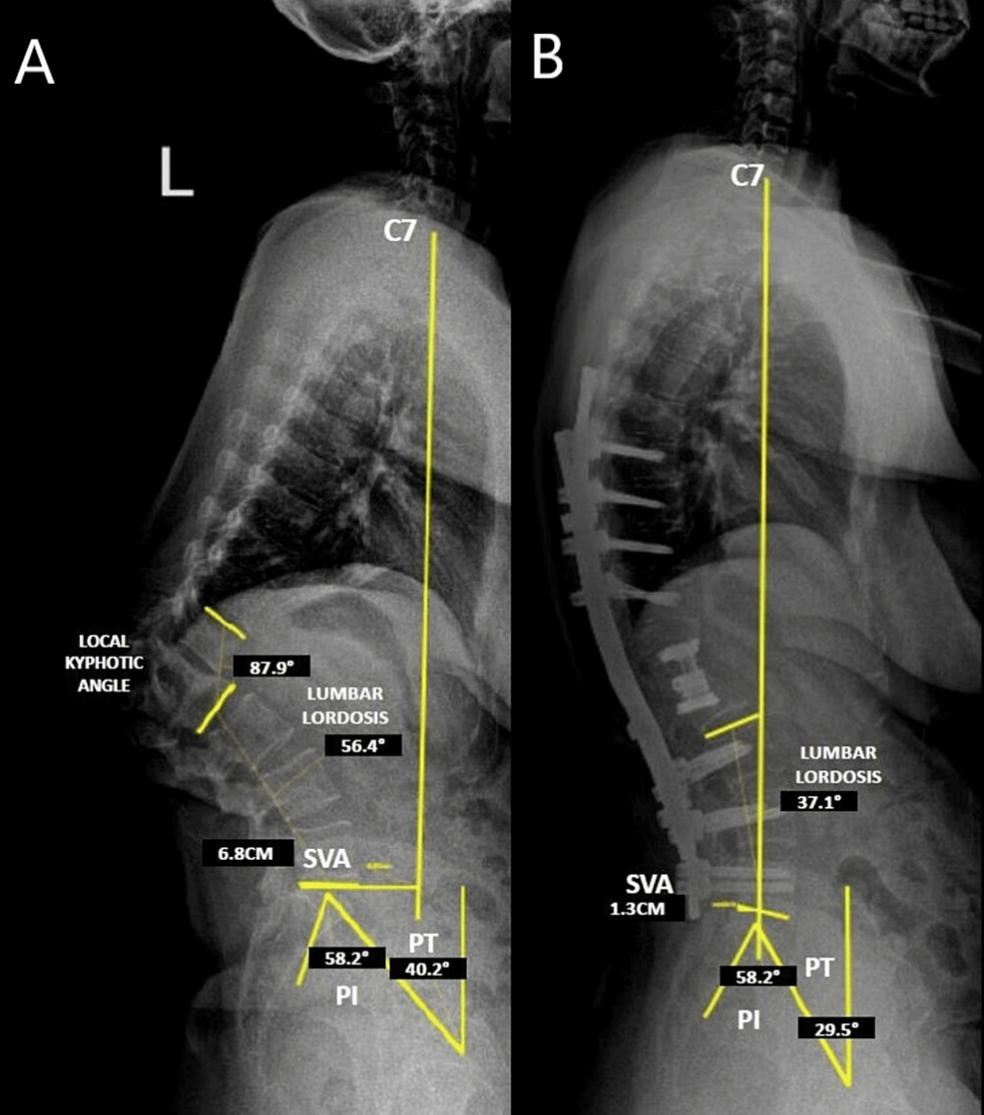
Preoperative (A) and postoperative (B) lateral whole-spine radiography for sagittal balance parameters.

Sagittal balance in severe kyphosis is a concern; fortunately, we were able to correct the patient’s sagittal vertical axis from 6.8 cm to 1.3 cm with a single posterior approach. The patient’s postoperative lumbar lordosis improved to 37° from 56.4° preoperatively. We were also able to reduce the pelvic tilt from 40.2° to 29.5° (Figure 5).

## Conclusions

Severe kyphosis is relatively rare. Our patient presented with various symptoms and therefore required surgical intervention. A standalone posterior approach with cage insertion and bone grafting can give good postoperative clinical outcomes and sagittal balance as well as fewer postoperative complications for patients with severe kyphoscoliosis.
